# Data on the characterization of non-cytotoxic pyomelanin produced by marine *Pseudomonas stutzeri* BTCZ10 with cosmetological importance

**DOI:** 10.1016/j.dib.2018.04.123

**Published:** 2018-05-04

**Authors:** Noble K. Kurian, Sarita G. Bhat

**Affiliations:** Department of Biotechnology, Cochin University of Science and Technology, Kochi 22, Kerala, India

## Abstract

The article focuses on data dealing with characterization of black brown melanin produced by marine bacteria *Pseudomonas stutzeri* BTCZ10. Figures deal with the production of melanin by strain BTCZ10 and characterization of the pigment using biophysical techniques. Table presents the data on photo-protective ability of melanin when blended with commercial sunscreens.

**Specifications Table**

**Table:** 

Subject area	*Biotechnology*
More specific subject area	*Pigment Biology, Bacterial Melanins*
Type of data	*Table (SPF data), Figures ( FT-IR, Proton NMR, EPR, TGA, Phase contrast microscopy)*
How data was acquired	FT-IR (Thermo Nicolet, Avatar 370),
	NMR (Bruker Avance III, 400 MHz),
	EPR (JEOL Model JES FA200),
	TGA (Perkin Elmer, Diamond TG/DTA),
	Microplate reader (ErbaLisaScan II)
Data format	*Analyzed (Statistical significance of the experiments was determined by one-way ANOVA using GraphPad Prism Software. At 95% confidence interval, p<0.05 were considered to be significant)*
Experimental factors	*Sun Protection factor and Cytotoxicity*
Experimental features	*Production of melanin, Characterization of melanin using FT-IR, NMR EPR and DPPH assay, Exploring melanin metabolism using inhibitors Kojic acid and Sulcotrione, Evaluating the Sun Protection Factor of melanin. Cytotoxicity of melanin exploration using MTT assay.*
Data source location	*Bacteria isolated from 96 m depth Grab samples (9°6’N, 75° 22’ E) at Arabian Sea*
Data accessibility	*Provided with this article*

**Value of the data**

• Data explores the biophysical characteristics of bacterial melanin which will help in understanding the structural and functional characteristics of the pigment.
• The data presented deals with the Sun Protection Factor (SPF) enhancement by bacterial melanin when blended with commercial sun screens; SPF enhancement is of great cosmetological importance.
• The cytotoxicity data of melanin gives insight into the utility of the pigment for more applications.

## 1 Data

**Fig. 1:**
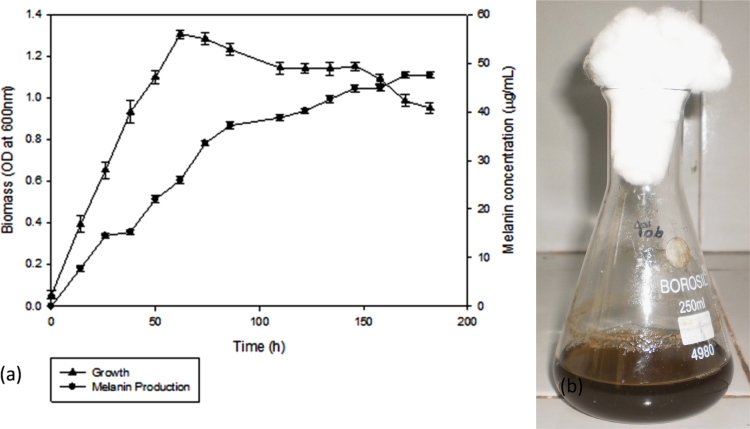
Production of Melanin (a) Time course of melanin production by *Pseudomonas stutzeri* strain BTCZ10 (b) Tyrosine basal broth after 180 h of BTCZ10 melanin production.

**Fig. 2:**
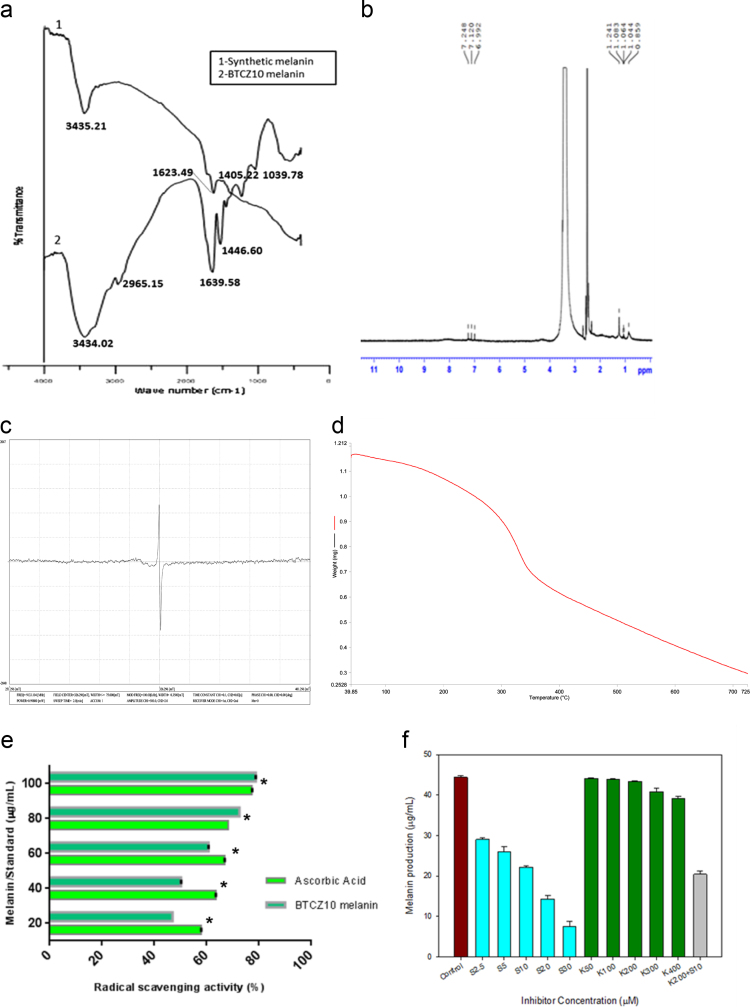
Spectral and biological characterization of melanin (a) FT-IR spectrum (b)^1^H NMR spectrum (c) EPR spectrum (d) TGA spectrum (e) DPPH radical scavenging potential (*p<0.05) (f) Effect of inhibitors on melanin production (K-Kojic Acid, S-Sulcotrione).

**Fig. 3:**
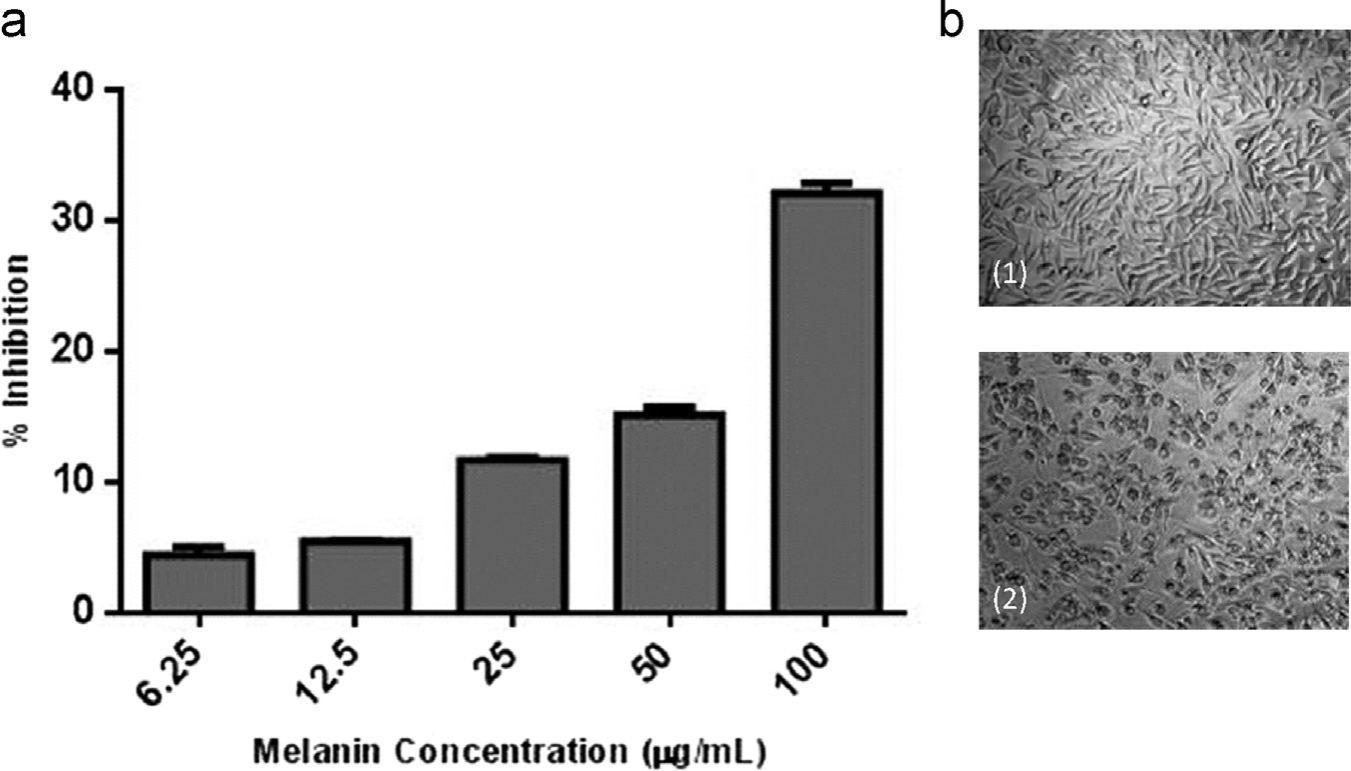
Cytotoxicity of melanin (a) Percentage of inhibition of L929 cell growth (b) Phase contrast micrograph of L929 cells (20× magnification) (1) Control (2) Treated.

The data describes the production of melanin by *Pseudomonas stutzeri* BTCZ10 (Fig. 1) and its biophysical characterization (Fig. 2). Sun Protective factor enhancement by the bacterial pigment is tabulated (Table 1). Dataset on cytotoxicity is presented (Fig. 3).

**Table 1:** Sun protection factor of melanin.

Commercial sunscreen	SPF value stated by the manufacturer	SPF value determined empirically during the current study	+BTCZ10 Melanin (0.005% w/w) SPF
Sunscreen 1	15	14.24 ± 0.007	16.72 ± 0.06
Sunscreen 2	15	14.61 ± 0.01	17.01 ± 0.02
Sunscreen 3	15	14.77 ± 0.05	17.10 ± 0.03
Sunscreen 4	17	16.47 ± 0.04	18.95 ± 0.01
Sunscreen 5	30	26.26 ± 0.04	28.18 ± 0.06

## 2 Experimental design, materials and methods

### 2.1 Experimental materials

Kojic Acid and Sulcotrione were purchased from Sigma Aldrich, USA. All other chemicals used were analytical grade from Himedia chemicals, India. L929 mouse fibroblast cell lines utilized for cytotoxicity evaluation were maintained in Dulbecco's modified eagles media (Himedia, India) supplemented with 10% FBS (Fetal Bovine serum) (Invitrogen, USA) and grown to confluence at 37 °C at 5% CO_2_ in a CO_2_ incubator (Eppendorf, Germany).The melanin producing *Pseudomonas stutzeri* BTCZ10 was isolated from 96 m depth grab samples collected during Sagar Sampada Cruise #305 from Arabian Sea (9°6′N, 75° 22′ E).

### 2.2 Production, extraction and purification of melanin

The bacteria was inoculated in tyrosine basal broth [1] and incubated at 37 °C for 180 h. Melanin production kinetics was studied by sampling at 12 h intervals and estimating bacterial growth (O.D 600 nm) and melanin production (O.D 400 nm) spectrophotometrically. The production medium was then centrifuged (5000 g for 10 min) to remove the cell debris. Resultant cell free supernatant was acidified (using 1N HCl) to pH below 2, allowed to stand for a week to precipitate melanin. Later, the melanin precipitate was boiled for one hour and washed thrice with 0.1N HCl, followed by water. Thereafter, the precipitate was boiled with absolute ethanol for 10 min and kept at room temperature for a day; followed by two washes in ethanol and air dried to get purified melanin [2].

### 2.3 Spectral and biological characterization of melanin

The FT-IR spectrum of melanin was recorded at 4000–400 cm^−1^, resolution 4 cm^−1^ using a Thermo Nicolet, Avatar 370 spectrophotometer equipped with KBr beam splitter with DTGS (Deuterated triglycine sulphate) detector (7800–350 cm^−1^).

The ^1^H NMR spectra were obtained at 27.4 °C on Bruker Avance III, 400 MHz. The NMR conditions were as follows: spectral width, 8223 Hz; acquisition time, 3.98 s; recycle delay, 2 s; and number of scans, 64.

EPR spectrum of melanin was obtained with JEOL Model JES FA200(X-Band) EPR spectrophotometer at spectral conditions included: frequency, 9.12 GHz; modulation frequency, 100.00 kHz; power, 0.99800 mW; field center, 326 mT; and sweep time, 2.0 min.

The thermal properties of the extracted, purified melanin were examined by TGA instrument (Perkin Elmer, Diamond, USA). The sample was scanned from 40–930 ^o^C with a heating rate of 10 ^o^C min^−1^.

Free radical scavenging activity of melanin was determined using 2, 2-diphenyl-1-picrylhydrazyl (DPPH) radical scavenging assay [3].

For melanin metabolism evaluation, inhibitors like Kojic acid (50–400 μM) and Sulcotrione (5–30 μM) and the combination of both were added to the melanin production medium [1]. And melanin production was monitored spectrophotometrically at 400 nm.

### 2.4 Sun Protection Factor of melanin

Sun Protection Factor (SPF) of melanin blended with commercial sunscreens was estimated by a modified protocol of Suryawanshi et al., 2015 [4]. Commercial sunscreens of 0.1 g were added to 10 mL of absolute ethanol. Melanin was added at a concentration of 0.005% to this mixture. Absorbance of the mixture in the UV range (290–320 nm) was quantified at 5 nm intervals using ethanol as the blank.

SPFs were calculated, according to Mansur et al. [5], using following formula,

$$\mathrm{SPF}=\mathrm{CF}\times \sum_{290}^{320}\mathrm{EE}(\lambda )\times I(\lambda )\times \mathrm{Abs}(\lambda )\;$$

where CF (correction factor) = 10; EE (λ) = erythmogenic effect of radiation with wavelength k; Abs (λ) = spectrophotometric absorbance value of the solution; and I = solar intensity spectrum. EE(λ) ×I is constant and was determined by Sayre et al. [6].

### 2.5 Cytotoxicity of melanin

Different concentrations (6.25, 12.5, 25, 50 and 100 μg/mL) of melanin were added to L929 cells and incubated for 24 h. The percentage difference in viability was determined by standard 3-(4, 5 dimethythiazol-2-yl)-2, 5-diphenyl tetrazolium bromide (MTT) assay [7] after 24 h of incubation.
