# Supplementary material for: Data on the characterization of non-cytotoxic pyomelanin produced by marine Pseudomonas stutzeri BTCZ10 with cosmetological importance
Source: Data Brief. 2018 May 4;18:1889–94. doi: 10.1016/j.dib.2018.04.123 (PMC5998704; doi:10.1016/j.dib.2018.04.123)
Supplement: Supplementary file 1 — Supplementary material [file mmc1.pdf]

CONFLICT OF INTEREST FORM

The authors declare that there are no known conflicts of interests associated with the data presented here and there has been no significant financial support for this work that could have influenced its outcome.

Signed by all authors as follows:

1	Noble K Kurian	Noble 19/4/18
2	Sarita G Bhat<br>(Corresponding Author)	Sarita Bhat 19/4/18
