# Supplementary material for: Data on the characterization of non-cytotoxic pyomelanin produced by marine Pseudomonas stutzeri BTCZ10 with cosmetological importance
Source: Data Brief. 2018 May 4;18:1889–94. doi: 10.1016/j.dib.2018.04.123 (PMC5998704; doi:10.1016/j.dib.2018.04.123)
Supplement: Supplementary file 2 — Supplementary material [file mmc2.docx]

**Data on the characterization of non-cytotoxic pyomelanin produced by marine *Pseudomonas stutzeri* BTCZ10 with cosmetological importance**

Noble K Kurian, Sarita G Bhat

Table: List of commercial SunScreens used with its ingredients

| **Commercial Sunscreen** | **Ingredients** |
| --- | --- |
| **Sunscreen 1**  **(Fair & Lovely^®^)** | Water, Palmitic acid, Stearic acid, Ethylhexylmethoxycinnamate, Laureth 23, Niacinamide, Butyl Methoxydibenzoylmethane, Glycerin, cetearyl ethyl hexandate & Isopropyl myristate, Hydroxystearic acid, Sodium ascorbylphosphate, Tocopheryl acetate, Allantoin, Pyridoxinehydrochlroride, Cetyl alcohol, Dimethicone, Acrylates/steareth-20, Methacrylate co polymer, Titanium di oxide & Aluminum hydroxide & Stearic acid, Ammonium acryloyldimethyltaurate, Beheneth-25, Methacrylate cross polymer, Phenoxyethanol methyl paraben, Propyl paraben, Potassium hydroxide, Titanium di oxide & Dimethicone, Disodium EDTA CI77491 & Isopropyl titanium triostearate & thriethoxysilylethylpolymethylsiloxyethyl, Dimethicone, Perfume. |
| **Sunscreen 2**  **(Olay^®^ – Natural White)** | Water, Niacinamide, Cyclopentasiloxane, Glycerin ,Polymethylsilsesquioxane, Dimethicone, Butylene glycol, Stearic acid, Ethyl hexyl methoxycinnamate, Titanium di oxide, Isohexadecane, Benzyl alcohol, Panthenol, Tocopherylactetate carbomer, Dimethiconol, Methylparaben, cetyl alcohol, Phenyl benzimidazole sulfonic acid, Sodium hydroxide, Aluminum starch, Octenylsuccinate, propylparaben, Acrylate /C10-30-alkyl acrylate cross polymer, Cetearyl alcohol, Cetearylglucoside, Cetyl palmitate, PEG100 stearate, Fragrance, Isostearic acid, Disodium EDTA, Menthol, Linalool, Hydroxyl isohexyl-3-cyclohexene, Carboxaldehyde, Butyl phenyl methyl propional, Benzyl salicylate, Citronellol, Alpha isomethyl ionone,CI16035,CI19140. |
| **Sunscreen 3**  **(Ponds^®^ – White Beauty)** | Water, Palmitic acid, Stearic acid, Niacinamide, Glycerol, Dimethicone, Ethylhexylmethoxycinnamate, Butyl methoxydibenzoyl methane, Titanium dioxide, Sodium ascorbyl phosphate, isopropyl myristate, Tocopherylacetate, Allantoin, Pyridoxylhydrochloride, Cetyl alcohol, Titanium dioxide & Aluminum hydroxide & Stearic acid, Phenoxyethanol, Methylparaben, Propylparaben, Potassium hydroxide, Disodium EDTA, CI14700, Sodium starch octenylsuccinate & Zea mays (Corn), starch & Lycopene & Glucose & Sodium ascorbate & Tocopherol ,Bh+,perfume, Linalool, Limonene, Butyl phenyl methyl propinal, Hexylcinnamate, citronellol, Alpha isolmethyl ionone, Geraniol, Hydroxyisohexyl-3-cyclohexene carboxyaldehyde, Benzyl benzoate. |
| **Sunscreen 4**  **(Garnier^®^ – White Complete)** | Water, Ethylhexylmethoxycinnamate, glycerin, Isohexadecane, Cyclohexasiloxane, Ammonium polyacryloyldimethyltaurate, Pentaerythirityl tetraethyl hexanoate, Titanium di oxide, PEG-100 stearate, Glyceryl stearate, Diethylamino hydroxyl benzoyl hexyl benzoate, Synthetic wax, Aluminum hydroxide, Ascorbylglucoside, Benzyl salicylate, Biosaccharide gum-1, Caprylol salicylic acid, Caprylol glycol, Cetyl alcohol, CI77891, Citric acid, lemon fruit extract, Ethyl paraben, Geraniol, Limonene, Linalool, Methyl paraben, Octyldodecanolphenoxy Ethanol, phenyl ethyl resorcinol, Potassium acetyl phosphate, silica, stearic acid, Tetrasodium EDTA, Fragrance (B38567/1). |
| **Sunscreen 5**  **(Suncote® Gel)** | Water, Octylmethoxycinnamate, Triethanol amine, Avobenzone, Phenyl benzimdazolesulphonic acid, Isononylisononanoate, Acrylates/C10-30 Alkyl Acrylates cross polymer, Tocopheryl Acetate, Diazolidinyl Urea, DMDM Hydrantoin, Perfume, BHT, CI16255. |
